# Supplementary material for: Giant modulation of the electronic band gap of carbon nanotubes by dielectric screening
Source: Sci Rep. 2017 Aug 18;7:8828. doi: 10.1038/s41598-017-09372-1 (PMC5562844; doi:10.1038/s41598-017-09372-1)
Supplement: Supplementary file 1 — Supplementary Information [file 41598_2017_9372_MOESM1_ESM.pdf]

Supplementary Information:
Giant modulation of the electronic band gap of carbon nanotubes by dielectric screening

Lee Aspitarte[1], Daniel R. McCulley[1], Andrea Bertoni[2], Joshua O. Island[3], Marvin Ostermann[3], Massimo Rontani[2], Gary A. Steele[3] and Ethan D. Minot[1*]

[1] *Department of Physics, Oregon State University, Corvallis, OR, 97331, USA*

[2] *Istituto Nanoscienze-CNR, Via Campi 213a, I-41125 Modena, Italy*

[3] *Kavli Institute of Nanoscience, Delft University of Technology, Lorentzweg 1, Delft 2628 CJ, The Netherlands*

Corresponding authors: ethan.minot@oregonstate.edu, aspitarl@oregonstate.edu

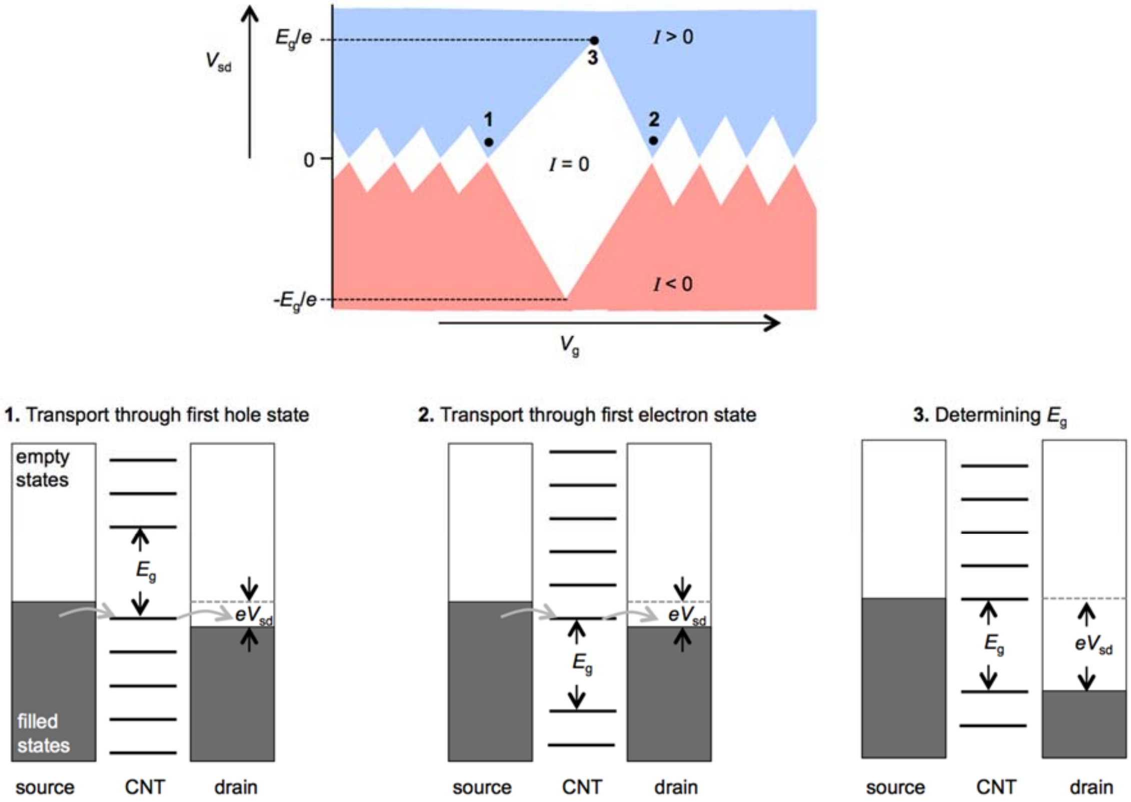

Figure S1. Analyzing low-temperature measurements of $I(V_g, V_{sd})$. (top) Blue represents positive current, red represents negative current, white represent no current. (bottom panels) The energy level diagrams illustrate the alignment of electron energy levels in the source electrode, the CNT and the drain electrode.

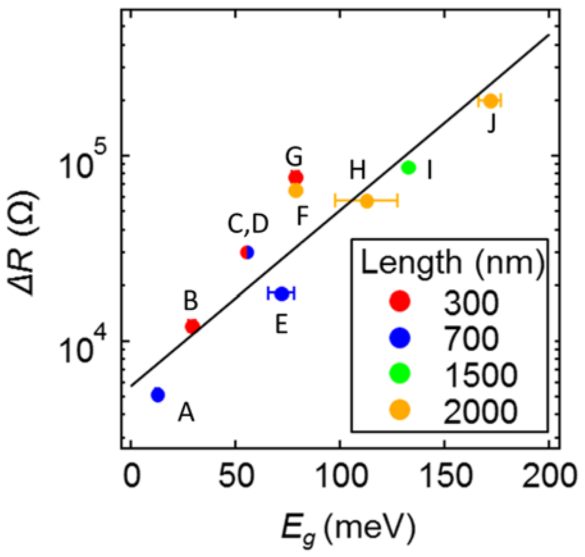

Figure S2. Summary of the Coulomb blockade measurements and corresponding device labels.

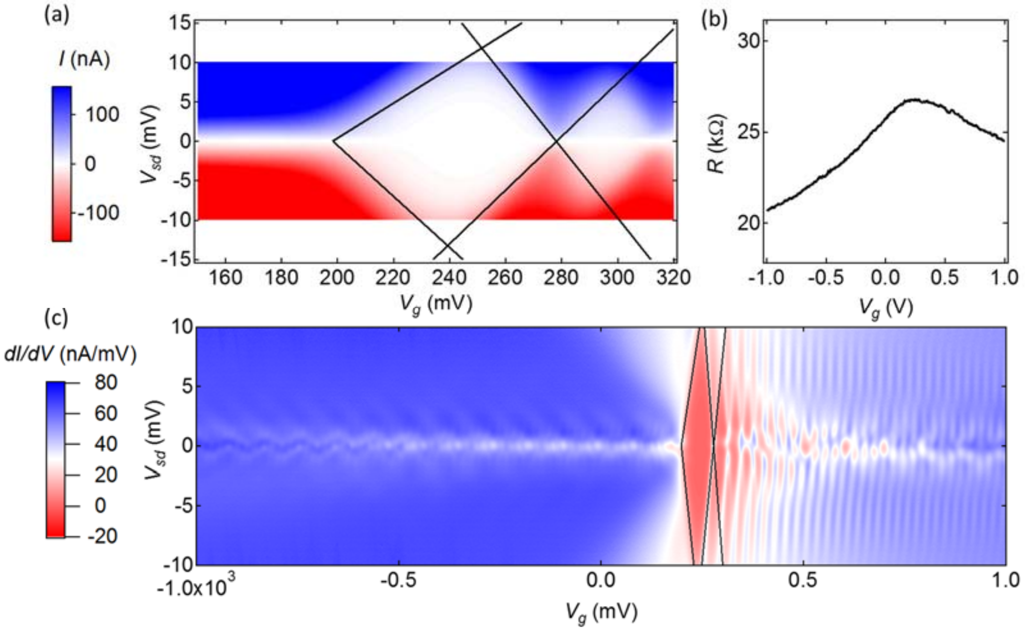

Figure S3. Transport measurements of Device A to determine E_g = 12.5 ± 0.7 meV, Δ*R* = 5.1 kΩ. The source-drain electrode separation is L = 700 nm. a) I(V_{sd},V_g) at T = 2 K. b) R(V_g) at 300K c) dI/dV_{sd} (V_{sd},V_g) at T = 2 K.

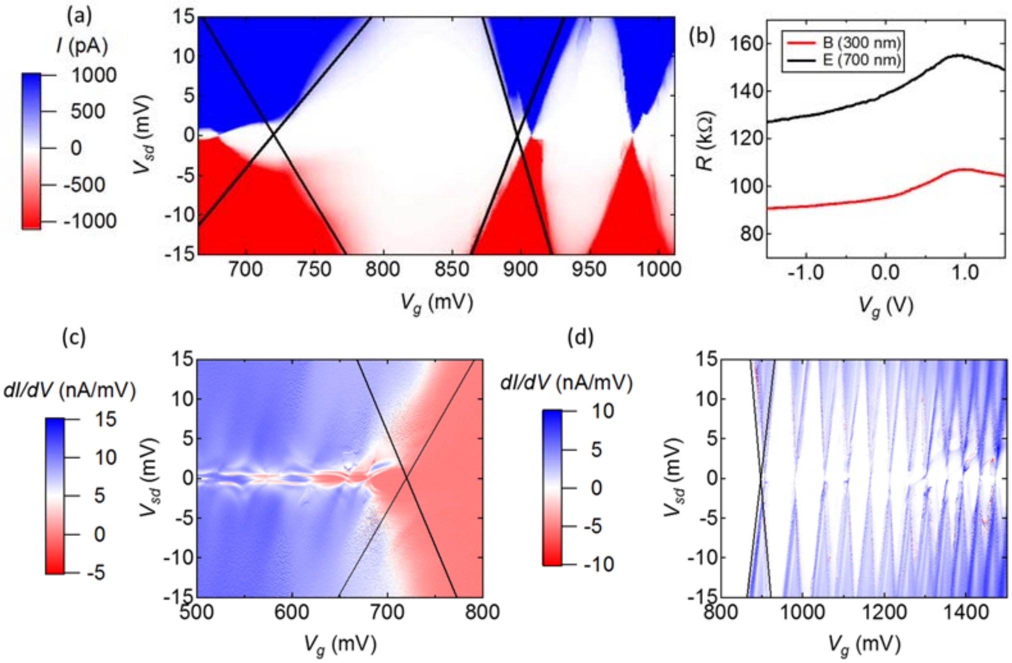

Figure S4. Transport measurements of Device B to determine E_g = 29.1 ± 1.7 meV, Δ*R* = 12.1 kΩ. The source-drain electrode separation is L = 300 nm. Device B (300 nm section) is nominally the same CNT as Device E (700 nm section). a) I(V_{sd},V_g) at T = 2 K. b) R(V_g) at 300K for both sections of the CNT. c) dI/dV_{sd} (V_{sd},V_g) at T = 2 K on the p-type side. d) dI/dV_{sd} (V_{sd},V_g) at T = 2 K on the n-type side.

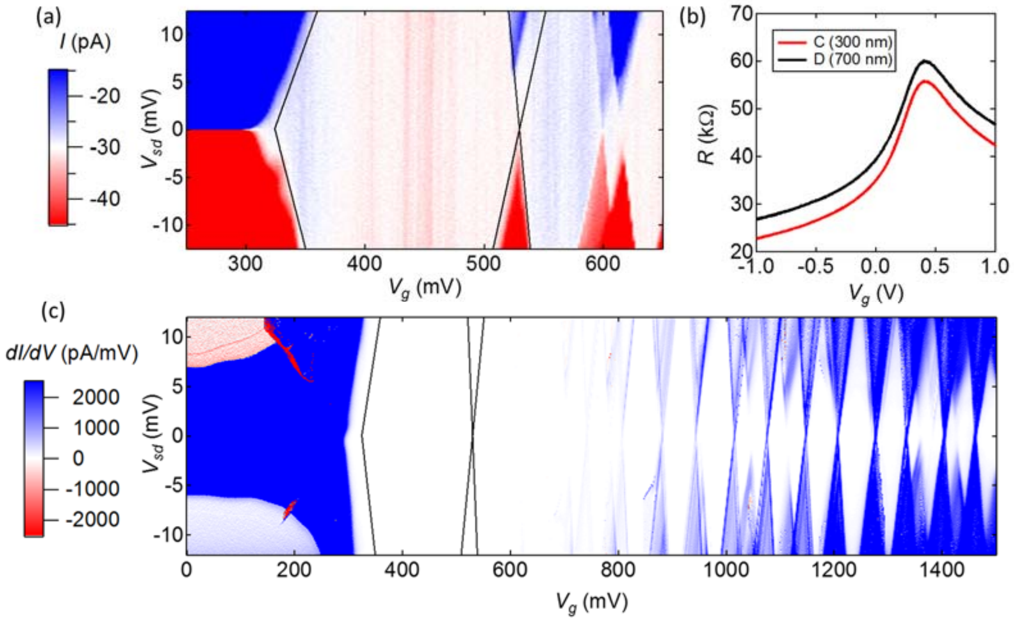

Figure S5. Transport measurements of Device C to determine E_g = 55.3 ± 1.7 meV, Δ*R* = 30.0 kΩ. The source-drain electrode separation is *L* = 300 nm. Device C (300 nm section) is the same CNT as Device D (700 nm section). a) I(V_{sd},V_g) at *T* = 2 K. b) R(V_g) at 300K for both sections of the CNT. c) dI/dV_{sd} (V_{sd},V_g) at *T* = 2 K.

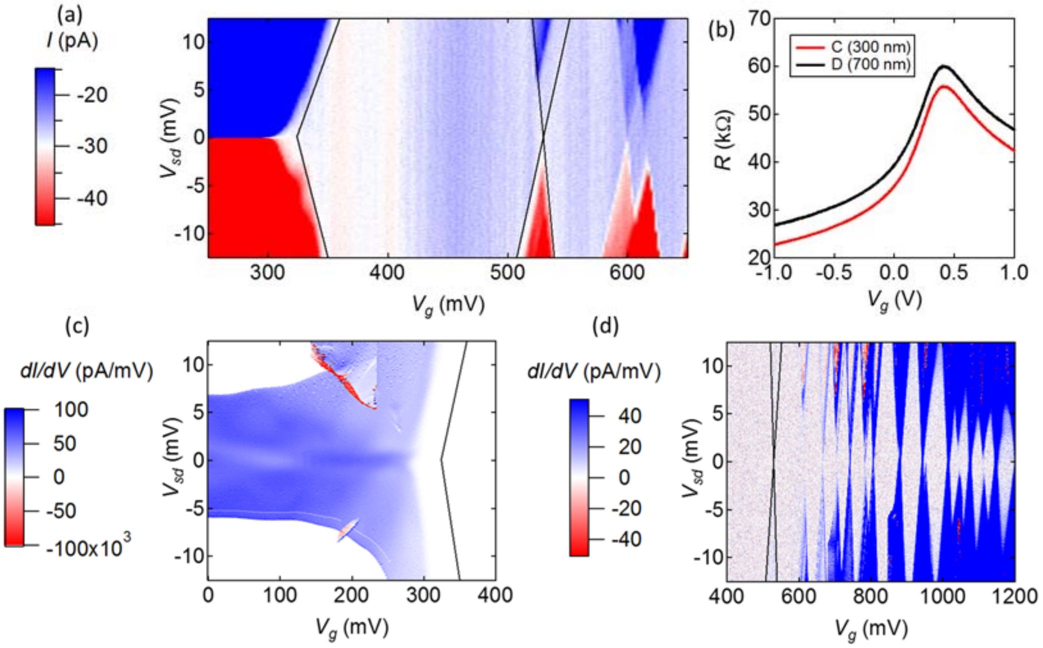

Figure S6. Transport measurements of Device D to determine E_g = 55.3 ± 1.7 meV, ΔR = 30.1 kΩ. The source-drain electrode separation is L = 700 nm. Device D (700 nm section) is the same CNT as Device C (300 nm section). a) $I(V_{sd},V_g)$ at T = 2 K. b) $R(V_g)$ at 300K for both sections of the CNT. c) $dI/dV_{sd}(V_{sd},V_g)$ at T = 2 K on the p-type side. d) $dI/dV_{sd}(V_{sd},V_g)$ at T = 2 K on the n-type side.

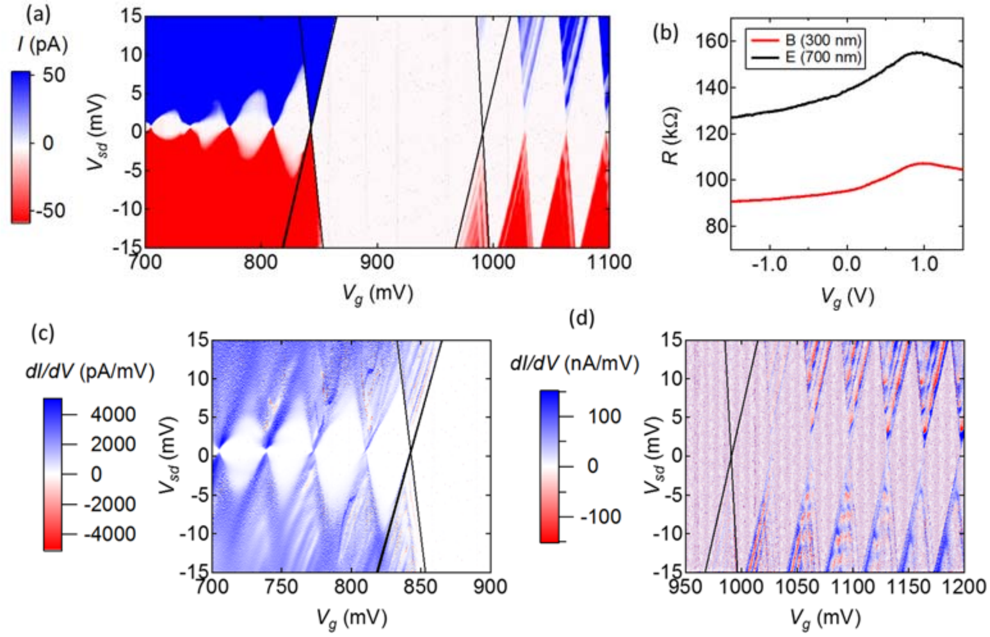

Figure S7. Transport measurements of Device E to determine E_g = 71.8 ± 6.2 meV, ΔR = 18.1 kΩ. The source-drain electrode separation is L = 700 nm. Device E (700 nm section) is the same CNT as Device B (300 nm section). a) $I(V_{sd},V_g)$ at T = 2 K. b) $R(V_g)$ at 300K for both sections of the CNT. c) $dI/dV_{sd}\,(V_{sd},V_g)$ at T = 2 K on the p-type side. d) $dI/dV_{sd}\,(V_{sd},V_g)$ at T = 2 K on the n-type side.

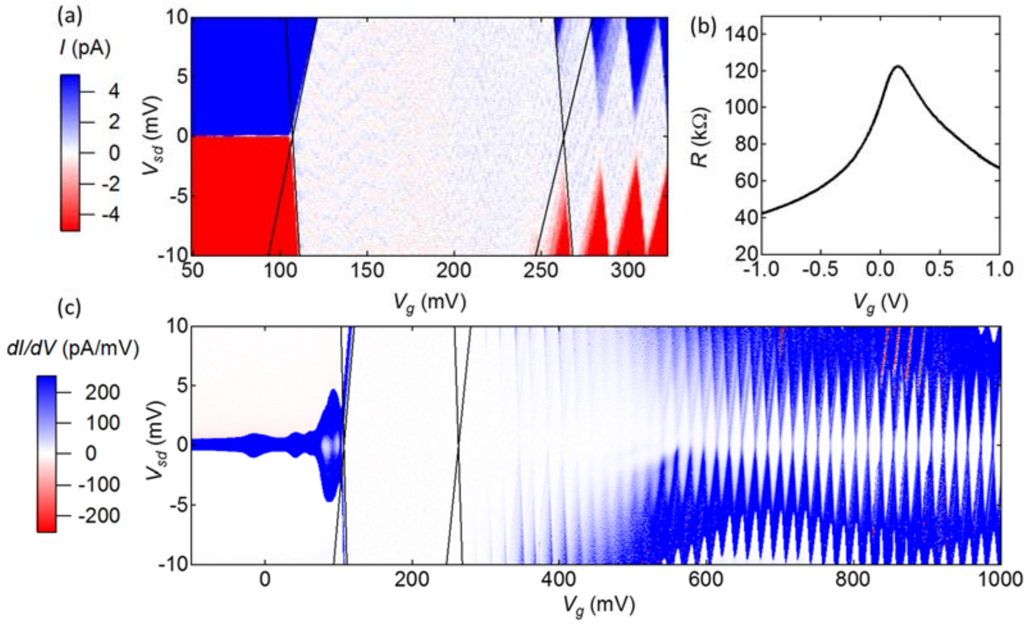

Figure S8. Transport measurements of Device F to determine E_g = 78.5 ± 2.0 meV, Δ*R* = 77.1 kΩ. The source-drain electrode separation is *L* = 300 nm. a) I(V_{sd},V_g) at *T* = 2 K. b) R(V_g) at 300K c) dI/dV_{sd} (V_{sd},V_g) at *T* = 2 K.

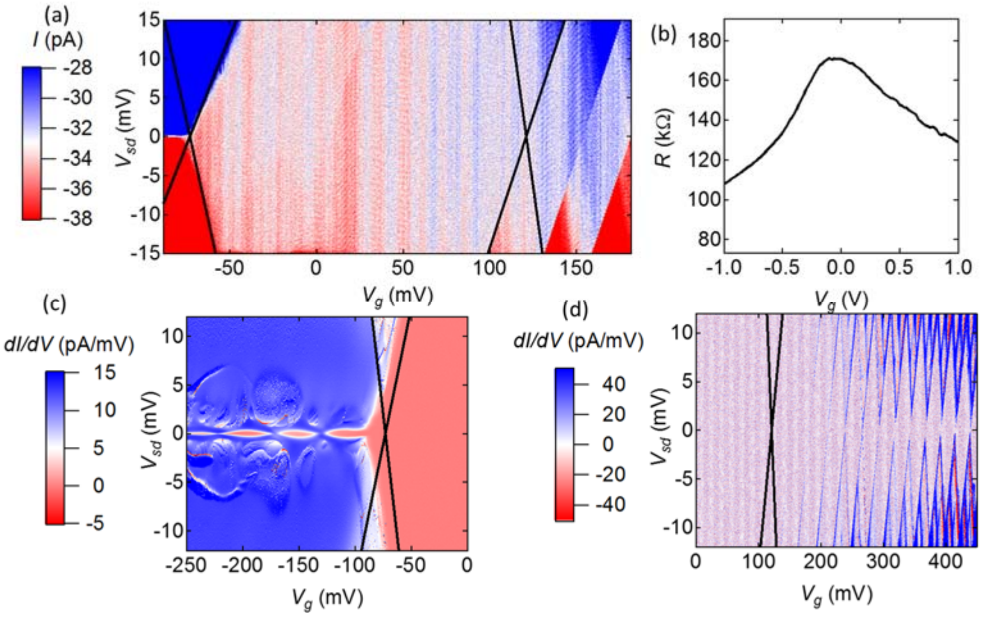

Fig. S9. Transport measurements of Device G to determine E_g = 78.6 ± 0.9 meV, Δ*R* = 65.5 kΩ. The source-drain electrode separation is L = 2000 nm. a) I(V_{sd},V_g) at T = 2 K. b) R(V_g) at 300K c) dI/dV_{sd} (V_{sd},V_g) at T = 2 K on the p-type side. d) dI/dV_{sd} (V_{sd},V_g) at T = 2 K on the n-type side.

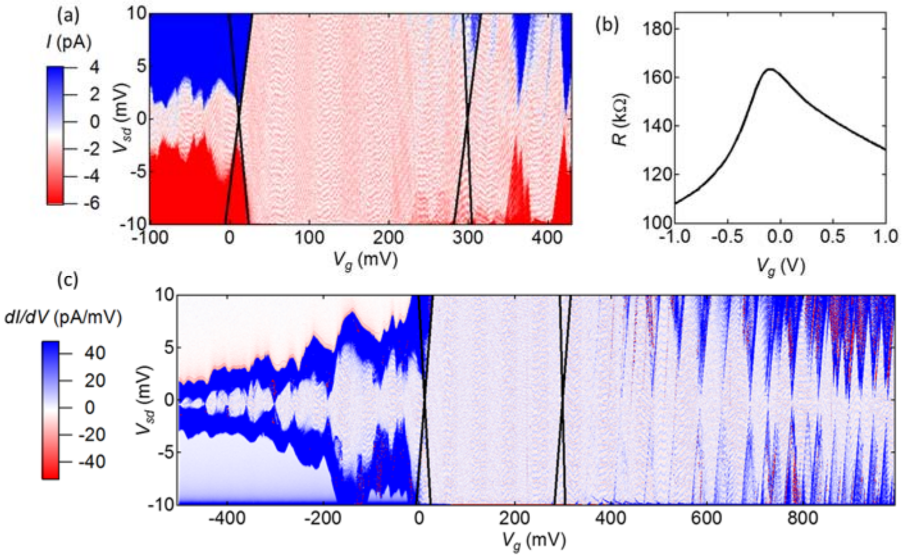

Fig. S10. Transport measurements of Device H to determine E_g = 112 ± 15 meV, Δ*R* = 57.6 kΩ. The source-drain electrode separation is *L* = 2000 nm. a) $I(V_{sd},V_g)$ at *T* = 2 K. b) $R(V_g)$ at 300K c) $dI/dV_{sd}\,(V_{sd},V_g)$ at *T* = 2 K.

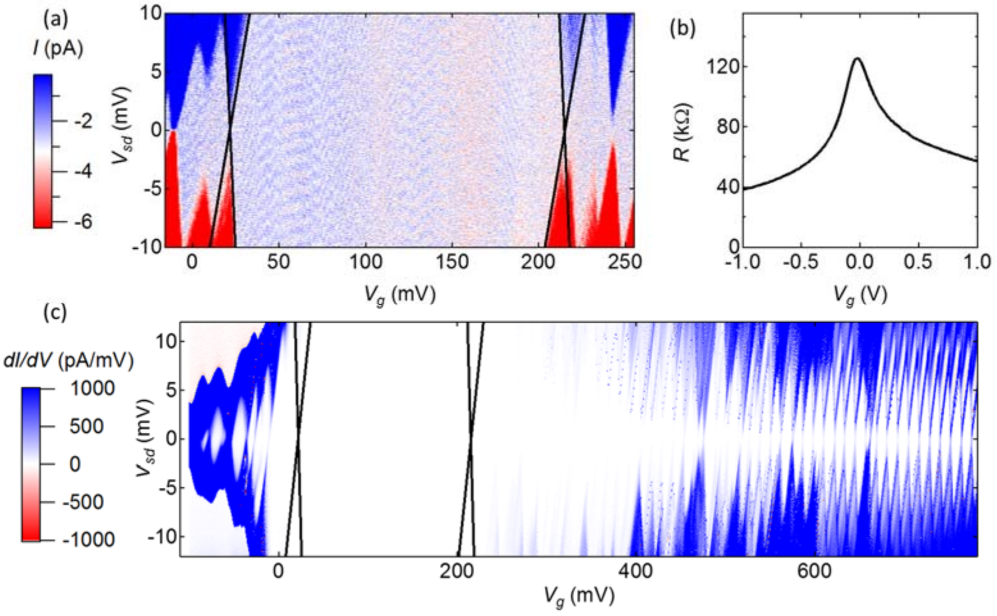

Figure S11. Transport measurements of Device I to determine E_g = 132 ± 0.1 meV, Δ*R* = 87.1 kΩ. The source-drain electrode separation is *L* = 1500 nm. a) I(V_{sd},V_g) at *T* = 2 K. b) R(V_g) at 300K c) dI/dV_{sd} (V_{sd},V_g) at *T* = 2 K.

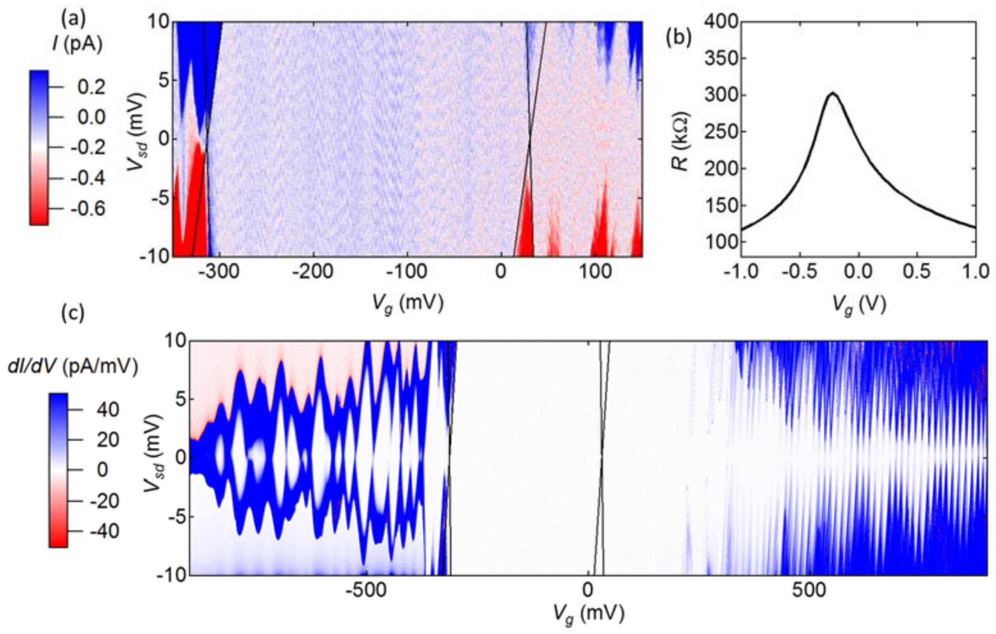

Figure S12. Transport measurements of Device J to determine E_g = 172 ± 5 meV, Δ*R* = 199 kΩ. The source-drain electrode separation is L = 2000 nm. a) I(V_{sd},V_g) at T = 2 K. b) R(V_g) at 300K c) dI/dV_{sd} (V_{sd},V_g) at T = 2 K.

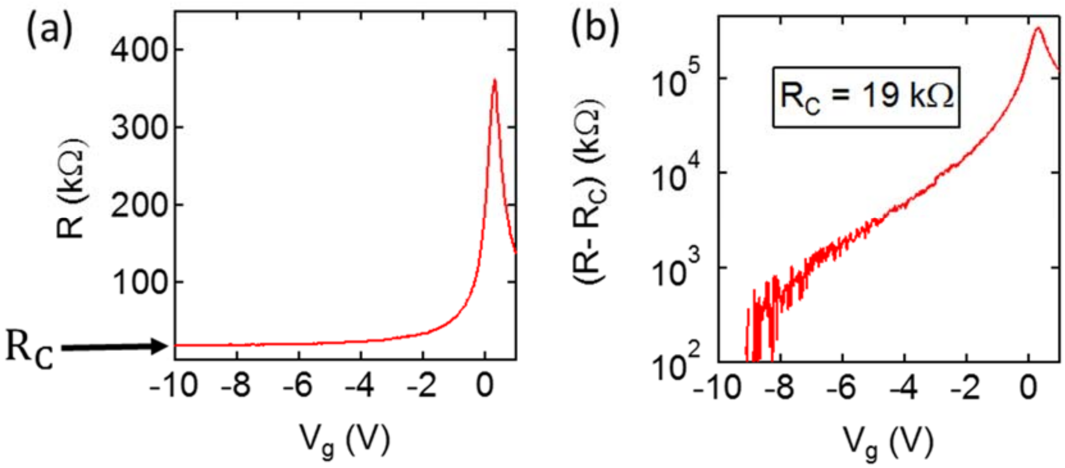

Figure S13. Determining R_c from room temperature $R(V_g)$ measurements. a) Room temperature $R(V_g)$. b) Log plot of $R(V_g)$-R_c demonstrating that $R(V_g)$ exponentially approaches R_c for large negative V_g.

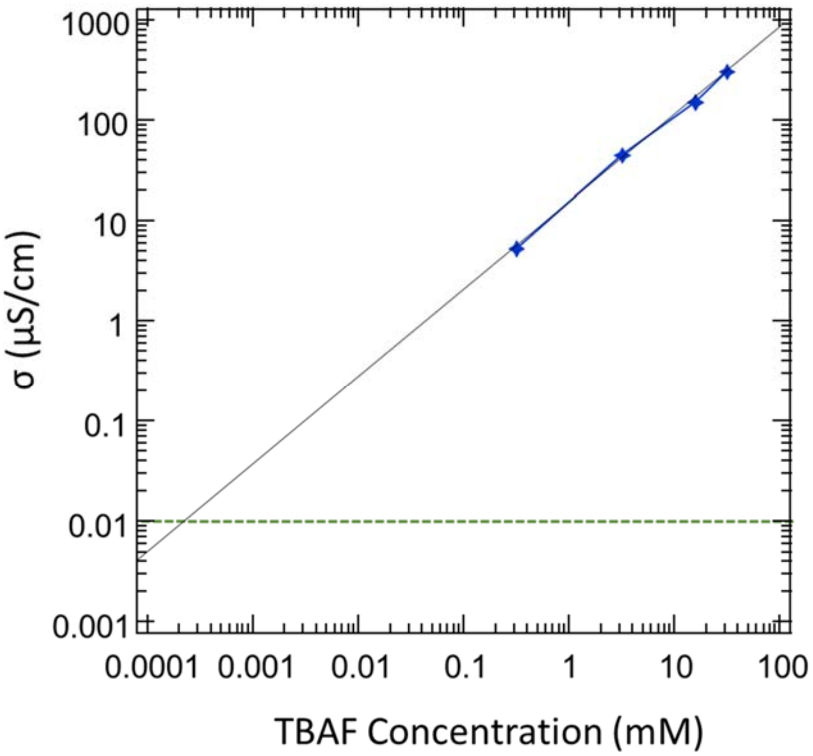

Figure S14. Log-log plot of isopropanol conductivity as a function of TBAF concentration. The gray line is a linear fit to the blue data point. The green dashed line is the residual conductivity of the pure isopropanol.

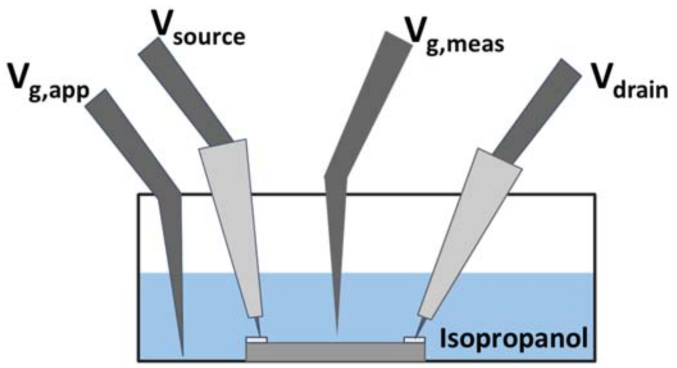

Figure S15. Schematic of the probe needles used for liquid-gating experiments. The source and drain probe-needles are coated in parylene-c.

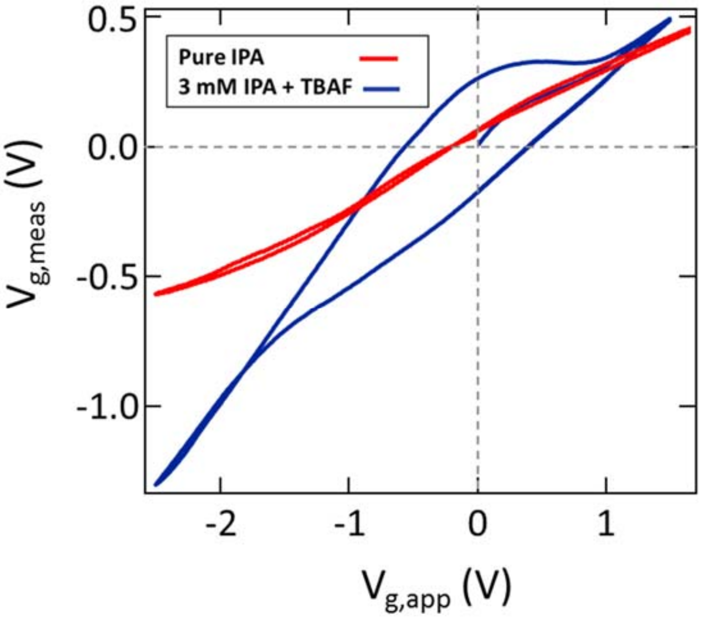

Figure S16. The potential measured in the fluid near the CNT, $V_{g,\mathrm{meas}}$, versus the potential applied to the fluid, $V_{g,\mathrm{app}}$. The addition of salt to the solution increases the slope but also introduces hysteresis.

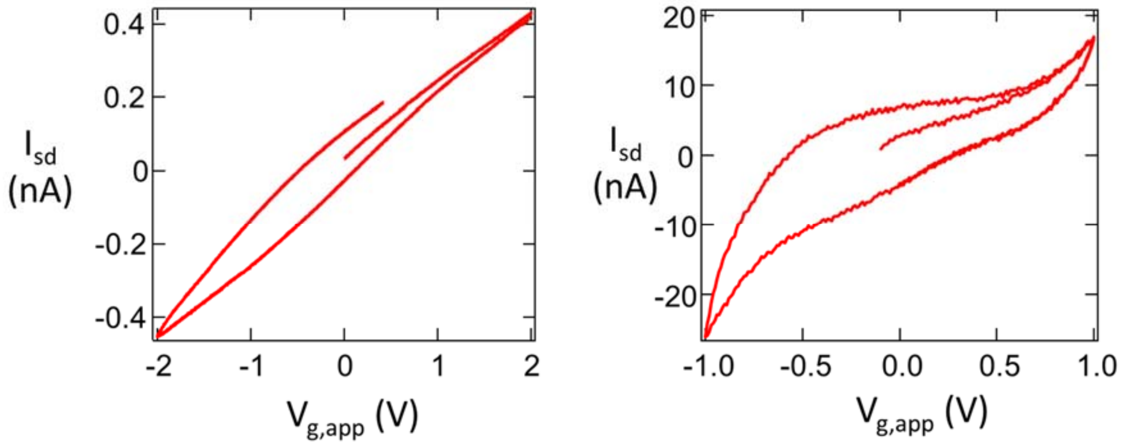

Figure S17. Faradaic leakage current measured from an electrode structure with no CNT. (left) Liquid gate applied via pure isopropanol. (right) Liquid gate applied via isopropanol with 3 mM TBAF.

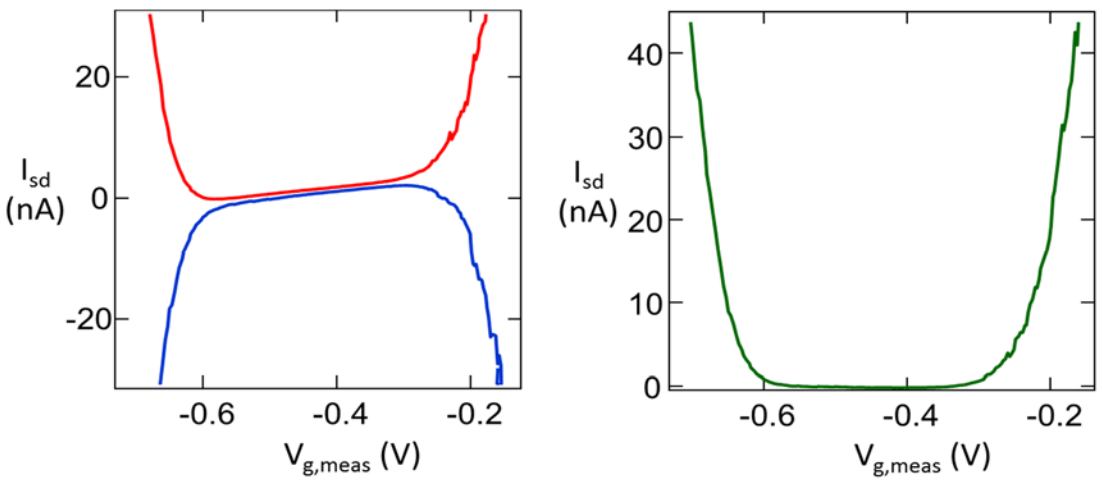

Figure S18. Distinguishing Faradaic currents from current flowing through a CNT. (a) The source-drain current, I_{sd}, is measured with V_{sd} = +50 mV (red curve) and V_{sd} = -50 mV (blue curve). When -0.55 V < $V_{g,meas}$ < -0.3 V, the CNT is not conducting and the measured currents are due to Faradaic leakage. The electrolyte is isopropanol with 95 mM TBAF. (b) Subtracting the blue curve from the red curve, and dividing by 2, reveals the current flowing through the CNT when V_{sd} = 50 mV.

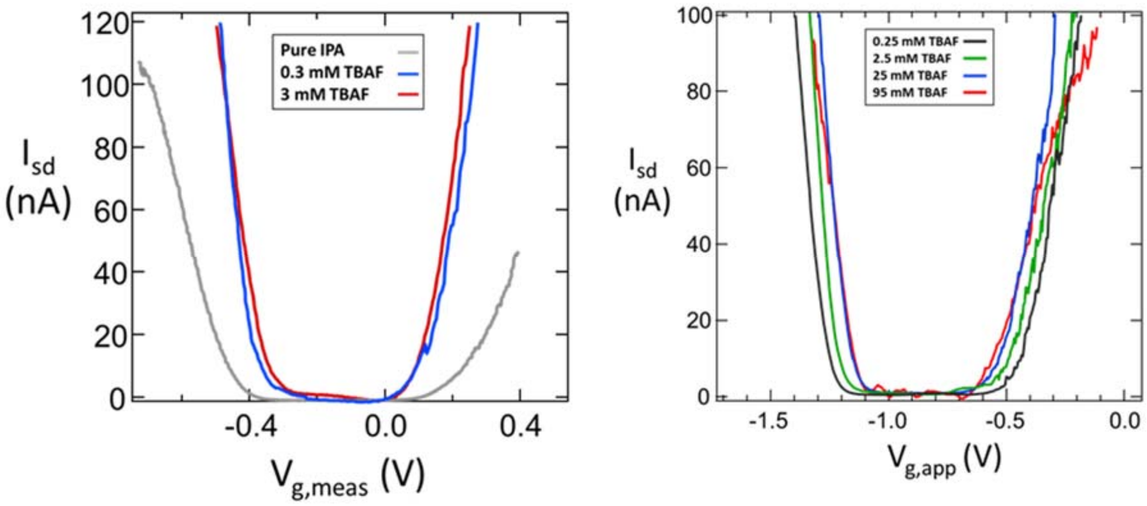

Figure S19. CNT device measured at various of salt concentrations. When salt concentration is sufficiently large, $I(V_{g,\mathrm{meas}})$ is independent of salt concentration.

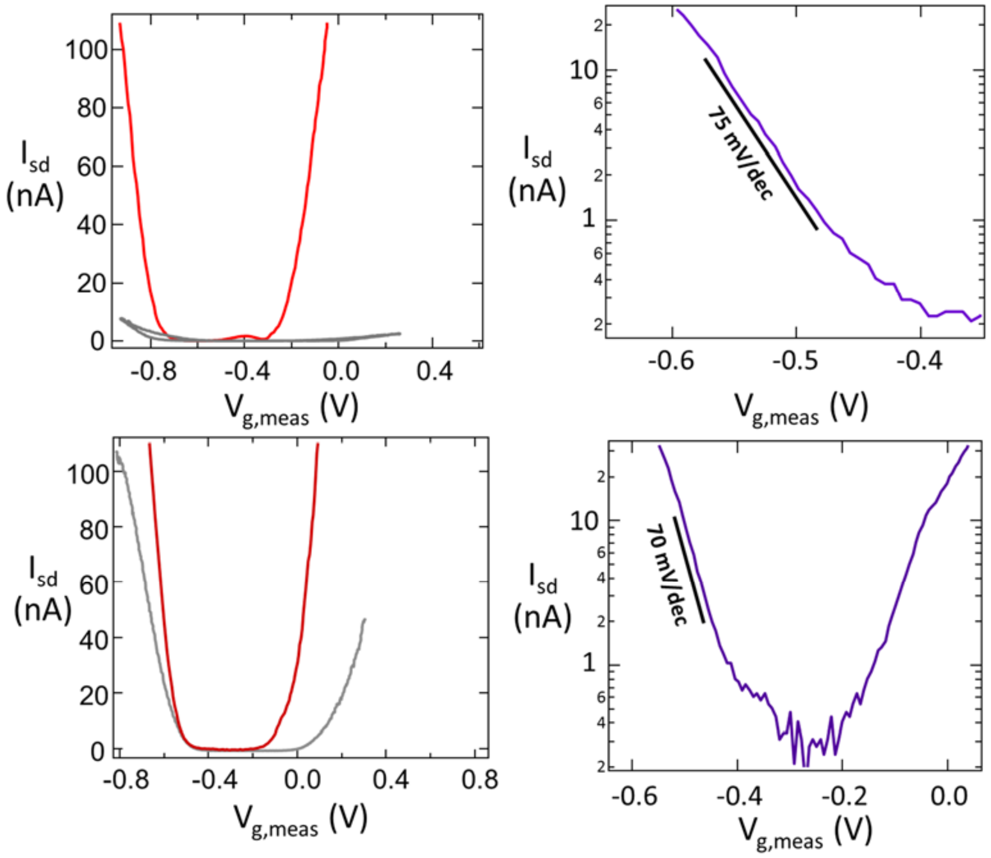

Figure S20. Liquid-gate measurement of device 13 (top) and device 14 (bottom). The grey curves are measured in pure isopropanol. The red curves are measured with 3 mM TBAF dissolved in isopropanol. The purple curves are log plots the data measured with 3 mM TBAF. The subthreshold slope is noted.

C (mM)	0.001	0.3	3	10	25	95
λ_D (nm)	500	8.4	2.7	1.5	0.9	0.5

Table S1. Debye screening lengths in IPA for monovalent salt.

Material	ϵ
Oxide	3.9
Air	1
Oil	3
IPA	18

Table S2. Relative permittivity of different dielectric materials of the device used in the self-consistent electrostatics simulations.

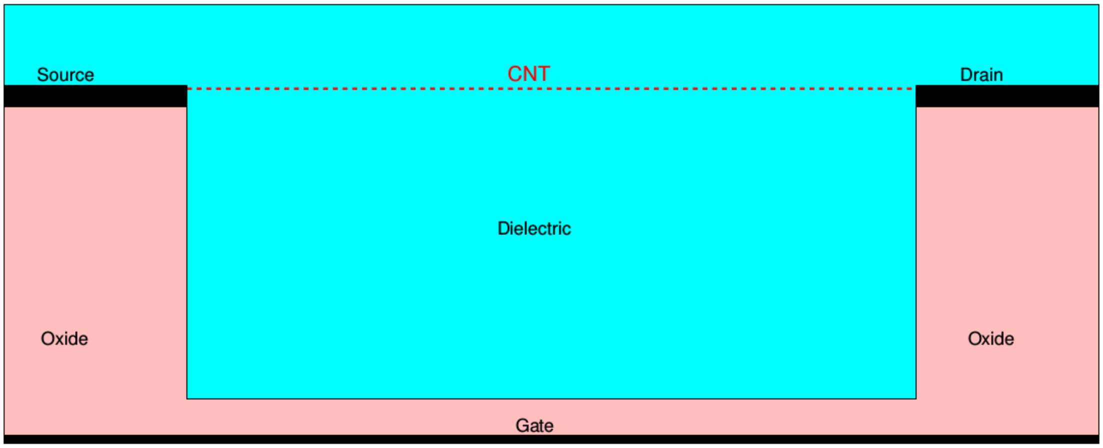

Figure S21. Scheme of the device representing the 3 μm × 1.2 μm simulation domain.

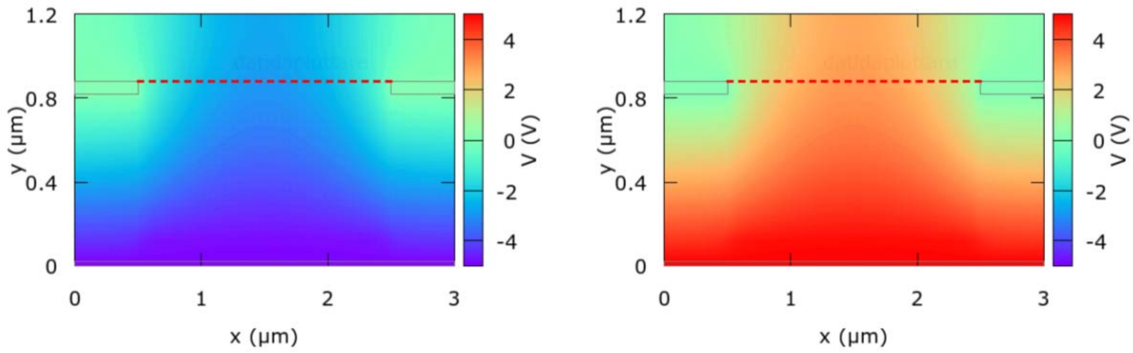

Figure S12. Electrostatic potential in the device with Air as the internal dielectric, at $\boldsymbol{V_{gate} = -5}$ V (left) and $\boldsymbol{V_{gate} = 5}$ V (right). The device structure is reported in Fig. 1 and the source and drain leads are at $V_{source} = 0$ and $V_{drain} = 25$ mV , respectively.

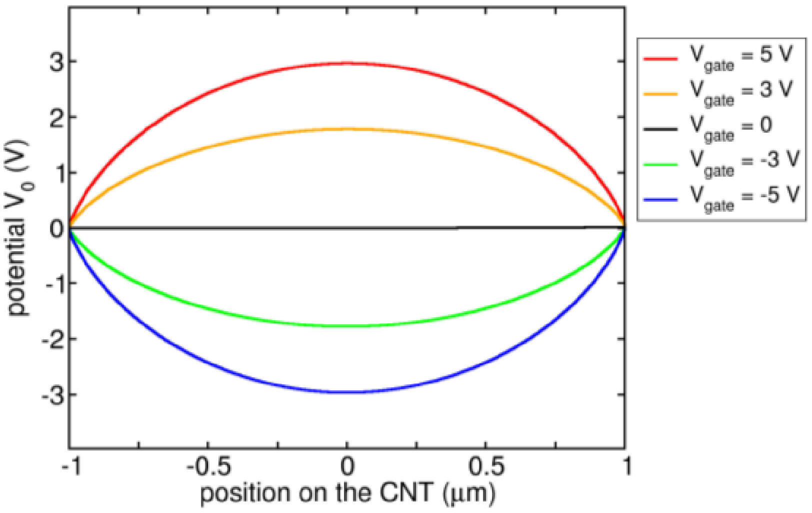

Figure S23. Electrostatic potential $\boldsymbol{V_0(x)}$ along the axis where the CNT will be placed. The environment is air. The five different V_{gate} values are indicated in the legend.

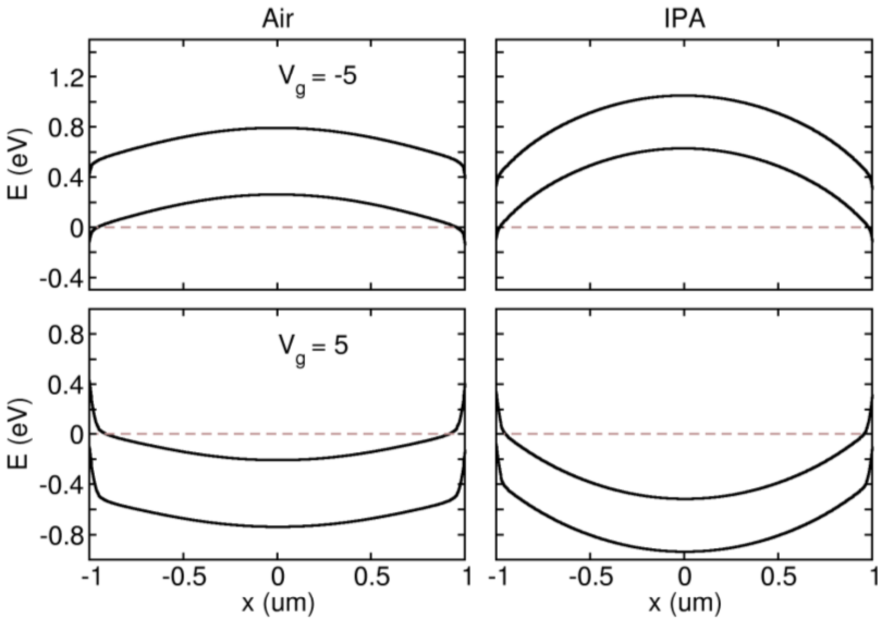

Figure S24. Self-consistent CNT bands of device #12 (see Table I of the main text) in Air (left graphs) and IPA (right graphs) with $\boldsymbol{V_{gate} = -5}$ V (top) and $\boldsymbol{V_{gate} = 5}$ V (bottom). Here Fermi level is pinned at $a = {}^{1}/_{4}$ from E_v and T= 300 K.

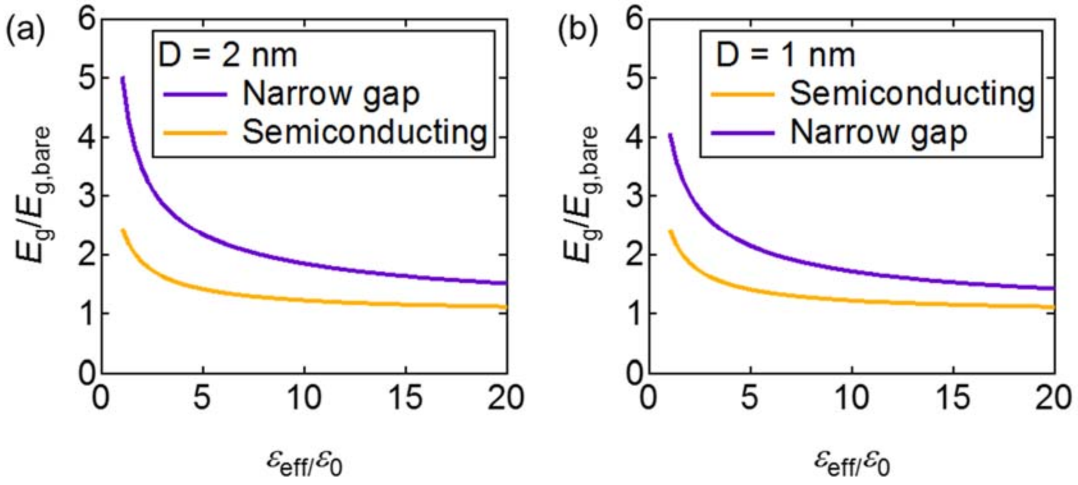

Figure S25. Enhancement of the transport gap, $E_g/E_{g,bare}$, as a function of the effective dielectric constant, ε_{eff}, for a narrow gap and semiconducting CNTs with diameter a) D = 2 nm and b) D = 1 nm. $E_g/E_{g,bare}$ depends on diameter for narrow-gap CNTs, but not semiconducting CNTs. $E_g/E_{g,bare}$ are calculated with the same method described in Figure 1 of the main text.

Supplementary Note 1 – Device Fabrication

Devices with a 2-μm source-drain separation were fabricated on 4-inch Si/SiO_2 wafers (500 nm oxide layer) on which gate electrodes were patterned and deposited (W/Pt 5 nm/60 nm). A layer of SiO_2 (800 nm thickness) was used to bury the gates. The source and drain electrodes were then patterned and deposited (W/Pt 5 nm/60 nm). Reactive ion etching was used to dig a trench between the source and drain electrodes. The Pt electrodes define the edge of the trench. CNT growth catalyst (1 nm Ti / 20 nm SiO2 / 1 nm Fe) was deposited on top of the source and drain electrodes. CNTs were grown using chemical vapor deposition in a tube furnace at 800C. The chips were shuttled in to avoid electrode degradation. The growth recipe consists of a 1 minute 1 SLM H_2 anneal followed by a 5 minute growth phase with 0.15 SLM ethanol, 0.3 SLM methanol, and 0.45 SLM H_2. The ethanol and methanol are introduced into Ar gas with a bubbler. Our process routinely yields ~ 10 CNT devices per die. Devices with source-drain separation L = 0.3 μm, 0.7 μm, and 1.5 μm were fabricated in a similar fashion, as described in Ref. 1.

Supplementary Note 2 - Coulomb blockade spectroscopy measurements

At the (V_g, V_{sd}) coordinates labeled 1 in Figure S1, there is a net flow of electrons from filled states in the source electrode to empty states in the drain electrode via the first hole state in the CNT. A similar situation occurs at higher Vg when current is transmitted through the first electron state (labeled as point 2). To find the transport band gap, Eg, we look for the (Vg, Vsd) coordinates at the peak of the largest diamond (point 3). The first electron state and the first hole state span the eVsd energy window and we have $E_g = V_{sd}/e$. We often make use of the straight edges of the Coulomb diamonds. If the maximum V_{sd} in the measurement range is less than E_g/e, point number 3 can be found by extrapolating the edges of the diamond. The gate coupling to the first hole state is sometimes different than the gate coupling to first electron state. In such cases the opposite edges of the diamond are not parallel.

Supplementary Note 3 – Liquid gate measurements

For liquid gating measurements, CNT devices are immersed in an isopropanol (IPA) solution that contains dissolved ions (TBA+ and F-). The ion content of the IPA is checked by measuring the conductivity of the liquid. Figure S14 shows conductivity as a function of concentration, $\sigma(C)$, for various TBAF concentrations. The residual conductivity of pure IPA is also shown (green dashed line). We estimate the residual concentration of dissolved ions in pure IPA to be < 0.001 mM.

The ion content of the liquid controls the Debye screening length, λ_D. By reducing λ_D, the Schottky barriers associated with the CNT-to-metal contacts become more transparent.[2] We estimate λ_D using the relationship

$$\lambda_D = \sqrt{\frac{\epsilon k_B T}{2e^2 N_A C}}, \qquad (S1)$$

where N_A is Avogadro's number, C is the salt concentration in mol/m^3, and ϵ is the dielectric constant of the liquid. Table 1 shows λ_D for various ion concentrations.

Figure S16 shows the relationship between $V_{g,\mathrm{meas}}$ and $V_{g,\mathrm{app}}$. The relationship is nearly linear at low salt concentration. At higher salt concentrations the coupling between $V_{g,\mathrm{app}}$ and $V_{g,\mathrm{meas}}$ is improved, but hysteretic effects are also apparent. When determining E_g (Fig. S20), it is important to plot conductance vs. $V_{g,\mathrm{meas}}$ rather than conductance vs. $V_{g,\mathrm{app}}$.

Electrical contact to the source and drain contacts on the CNT chip is made via parylene-c-coated tungsten probe needles (Microprobes for Life Science) that are positioned using x-y-z micromanipulators. The shaft of the probe needle is coated in a 3-micron-thick insulating layer of parylene-c. The insulating coating minimizes Faradaic leakage current between the source electrode, drain electrode and liquid. Only the tip of the metal needle (~ 500 μm^2) contacts the liquid. Voltage is applied to the liquid using a bare tungsten needle, $V_{g,\mathrm{app}}$. A second bare tungsten needle is used to measure the liquid potential, $V_{g,\mathrm{meas}}$ using a voltmeter with 10 GΩ input resistance.

To measure the Faradaic leakage current, we performed corol experiments on an electrode structure that has no CNT. Figure S17 shows a typical Faradaic leakage current between the liquid gate and the electrode structure.

When characterizing a CNT device, we use the following procedure to distinguish between current that passes through the CNT channel and Faradaic leakage currents. The device is first measured with V_{sd} = +50 mV (Fig. S18a, red curve), and then measured with V_{sd} = -50 mV (Fig. S18a, blue curve). By inverting the source-drain voltage, the current flowing through the CNT is reversed. However, the Faradaic current between the liquid gate and the grounded drain electrode is unchanged. Therefore, subtracting the blue curve from the red curve and dividing by 2 corresponds to the current flowing through the CNT (Fig. S18b).

The conductivity of the CNT device increases with salt concentration because the Schottky barrier contacts become more transparent. Figure S19 shows measurements taken at variety of salt concentrations. Faradaic leakage current has been subtracted. In Fig. 19a, the shape of the I-V_g curve stops changing at 25 mM TBAF. In Fig. 19b, the shape of the I-V_g stops changing at 3 mM TBAF. When determining E_g, we increase salt concentration until the shape of the I-V_g curve stops changing.

We performed liquid-gate measurements of E_g on three different CNT devices (see Table 1 of main text). Data from Device 13 and 14 are shown in Figure S21 (Device 12 is shown in the main text).

Supplementary Note 4 - Numerical simulations

The band profile of the CNT FET in Figure 2b is calculated by first solving the 2D Laplace equation without the presence of the CNT. Then, the charge density profile along the CNT is computed by means of a self-consistent cycle. Details of this process are described below.

Device structure and electrostatics.

The first step to model the band profile (Figure 2b) of the CNT FET device is the calculation of the electrostatic potential $V(x, z)$ generated on the CNT by the voltage

applied to the source, the drain and the gate metallic leads, when a dielectric with permittivity ϵ is present. This is achieved by solving the corresponding 2D Laplace equation

$$\frac{\partial}{\partial x}\left[\epsilon(x,z)\frac{\partial}{\partial x}\right]V(x,z)+\frac{\partial}{\partial z}\left[\epsilon(x,z)\frac{\partial}{\partial z}\right]V(x,z)=0$$

where $\epsilon(x,z)$ is the position-dependent relative permittivity of the material. Numerical values used in the simulations are reported in Table I.

The numerical solution is performed via a finite difference scheme on the 3 μm × 1.2 μm rectangular simulation domain shown in Fig. S21, where the device structure is also reported. The thickness of the gate in the z direction (growth direction) is 20 nm and the thickness of the source and drain is 60 nm. The gate oxide is 100 nm thick and the oxide layer below the source and drain leads is 700 nm plus the gate oxide, leading to a total distance of 800 nm between the gate and the source/drain leads. In the x direction, the source and drain are 500 nm wide, leading to a 2 μm gap between them, where the 2 μm long CNT acts as the channel of the FET device. A rectangular grid of 1200×800 points is used. The electric voltage of the metallic leads (in black in Fig. S21) are included by imposing Dirichlet boundary conditions on them, while Neumann conditions with zero normal derivative are imposed on the remaining boundaries. The linear system resulting from the finite difference discretization is solved numerically to obtain the electric potential distribution inside the domain. Finally, the potential $V_0(x)$ along the 1D segment representing the CNT (red dashed line in Fig. S21) is extracted.

As an example, Fig. S22 reports the potential profile induced on the device area by two gate voltage setups, namely $V_{gate} = -5$ V (left) and $V_{gate} = 5$ V (right), with $V_{source} = 0$ and $V_{drain} = 25$ mV in both cases.

Figure S24 shows $V_0(x)$ for the two cases of Fig. 2, together with $V_{gate} = -3$ V, $V_{gate} = 0$ V and $V_{gate} = 3$ V, in air.

Self-consistent charge and potential of wide-gap CNTs.

The charge density profile along the CNT is computed by means of a self-consistent cycle, with the CNT considered as a quasi-1D semiconducting system, where transverse

excitations are neglected due to the strong confinement. The computed behavior of the band profiles against the gate voltage and other device parameters (e.g. its geometry, effective dielectric permittivity and Fermi level pinning) is instrumental to the rationalization of the conductance characteristics of the CNT devices.

The CNT transport gap $E_{gap} = E_c - E_v$ is taken from the experimental estimation, as described in the main text, and the Fermi level μ of the unbiased system is set within the gap, at the source and drain leads. By inspecting the asymmetric current vs V_{gate} characteristic of the device (see Fig. 2d of the main text) it is clear that μ is closer to E_v than to E_c.[3] Thus, we set $\mu = E_v + aE_{gap}$ with $a < 0.5$. Specifically, a varies from $1/3$ to $1/10$ in different simulations. In each simulation, Fermi level is pinned at a fixed value throughout the self-consistent cycle.

The electrostatic potential $V_0(x)$ is then added to the conduction band and valence band energies along the CNT, leading to $E_0^c(x) = E_c - eV_0(x)$ and $E_0^v(x) = E_v - eV_0(x)$. Since the source-drain voltage bias is included in $V_0(x)$,the Fermi level of the drain must be shifted to $\mu - eV_{drain}$, while the Fermi level of the source is μ since $V_{source} = 0$. Within the self-consistent procedure, the electron and hole densities are obtained as[4]

$$n_e(x) = 2\sqrt{\frac{m_e^* k_B T}{2\pi\hbar^2}}\mathcal{F}_{-\frac{1}{2}}\left(\frac{\mu + E_0^c(x) + eV(x)}{k_B T}\right)$$

$$n_h(x) = 2\sqrt{\frac{m_h^* k_B T}{2\pi\hbar^2}}\mathcal{F}_{-\frac{1}{2}}\left(\frac{\mu - E_0^v(x) - eV(x)}{k_B T}\right)$$

where T is the temperature, $\mathcal{F}_{-\frac{1}{2}}$ is the complete Fermi-Dirac integral of order $-\frac{1}{2}$, and $V(x)$ is the self-consistent interaction potential of the carriers on the CNT: in the first iteration it is set to zero or to a best-guess function. The effective mass m_e^* (m_h^*) of electrons (holes) in the semiconducting CNT is obtained from the RPA approach described in Ref. [5] (with the inclusion of the effect due to tube curvature, as parametrized in Ref. [6]) by considering the experimental value of E_{gap}. We note however, that, contrary

to the effective dielectric permittivity, its variation is small and does not affect substantially our results.

From the charge distributions $n_e(x)$ and $n_h(x)$, the interaction potential is obtained via a simple Coulomb integration, where the integral is performed along the CNT and an effective permittivity for the CNT carriers in a given the dielectric environment is used. The latter is obtained from the approach described in Ref. [5] and Ref. [6], as the effective masses. A dumping factor is included to avoid the divergence of the kernel and accounts for the finite transverse extension of the CNT phenomenologically. Its value is kept fixed to $\gamma = R/4$, where R is the CNT radius. The solution of the set of equations is iterated until the relative change in both the electron and hole densities is less than 10^{-5} in any point of the CNT.

Figure S24 shows an example of self-consistent conduction band and valence band resulting from the simulations of device #12 of Table I of the main text. Simulations parameters are given in the caption. Note that a similar result, only for air, is shown in Fig. 2b of the main text, where Fermi level was pinned at $a = {}^{1}/_{10}\, E_{gap}$ from E_v and $V_{gate} = 3$ V. Schottky barriers at the contacts suppress n-type ($V_{gate} > 0$) conductance for both dielectrics.

Supplementary References

1. Schneider, B. H. *et al.* Coupling carbon nanotube mechanics to a superconducting circuit. *Sci. Rep.* **2,** 329–332 (2012).
2. Braga, D., Gutiérrez Lezama, I., Berger, H. & Morpurgo, A. F. Quantitative Determination of the Band Gap of WS2 with Ambipolar Ionic Liquid-Gated Transistors. *Nano Lett.* **12,** 5218–5223 (2012).
3. Léonard, F. *The physics of carbon nanotube devices*. (William Andrew, 2009).
4. Datta, S. *Quantum transport : atom to transistor*. (Cambridge University Press, 2006).
5. Ando, T. Excitons in carbon nanotubes. *J. Phys. Soc. Japan* (1997). at <http://jpsj.ipap.jp/link?JPSJ/66/1066/>

6. Laird, E. A. *et al.* Quantum transport in carbon nanotubes. *Rev. Mod. Phys.* **87,** 703–764 (2015).
